# Network-based support vector machine for classification of microarray samples

**DOI:** 10.1186/1471-2105-10-S1-S21

**Published:** 2009-01-30

**Authors:** Yanni Zhu, Xiaotong Shen, Wei Pan

**Affiliations:** 1 Division of Biostatistics, School of Public Health, University of Minnesota, Minneapolis, Minnesota 55455, USA; 2 School of Statistics, University of Minnesota, Minneapolis, Minnesota 55455, USA

## Abstract

**Background:**

The importance of network-based approach to identifying biological markers for diagnostic classification and prognostic assessment in the context of microarray data has been increasingly recognized. To our knowledge, there have been few, if any, statistical tools that explicitly incorporate the prior information of gene networks into classifier building. The main idea of this paper is to take full advantage of the biological observation that neighboring genes in a network tend to function together in biological processes and to embed this information into a formal statistical framework.

**Results:**

We propose a network-based support vector machine for binary classification problems by constructing a penalty term from the *F*_∞_-norm being applied to pairwise gene neighbors with the hope to improve predictive performance and gene selection. Simulation studies in both low- and high-dimensional data settings as well as two real microarray applications indicate that the proposed method is able to identify more clinically relevant genes while maintaining a sparse model with either similar or higher prediction accuracy compared with the standard and the *L*_1 _penalized support vector machines.

**Conclusion:**

The proposed network-based support vector machine has the potential to be a practically useful classification tool for microarrays and other high-dimensional data.

## Background

The past two decades have witnessed rapid advances in gene expression profiling with the microarray technology, which not only brighten the prospect of deciphering the complexity of disease genesis and progression at the genomic level, but also revolutionize the diagnostic, therapeutic, and prognostic approaches. Up to recently, diagnostic classification and prognostic assessment have been based on conventional clinical and pathological risk factors, such as patient age and tumor size, many of which are believed to be secondary manifestation [1]. The advent of microarray technology allows researchers to explore primary disease mechanisms by comparing gene expression profiles for malignant and normal cells. The regularity and aberration in the expression patterns of certain genes shed light on their functions and pathological importance [2]. Studies that seek to identify gene markers to refine diagnostic classification and improve prognostic prediction in the context of gene expression data have enriched the literature [3-5]. In recent years, researchers have realized that gene markers identified from microarrays drawn from difierent studies on the same disease across similar cohorts lack consistency [6,7]. A possibly more effective means to resolve this problem is to employ a network-based approach, that is, to identify markers as gene subnetworks, defined as groups of functionally related genes based on a gene network, instead of treating individual genes as completely independent and identical a priori as in most existing approaches [1]. A novel network-based approach proposed recently [1,8] can be summarized as follows: (1) randomly searching subnetworks and assigning a score to each subnetwork that characterizes the subnetwork-wise gene expression level; (2) identifying significant subnetworks that can well discriminate the clinical outcome; (3) constructing a classifier based on the significant subnetworks with a conventional statistical tool, such as logistic regression. Essentially such a network-based approach aggregates gene expression data at the subnetwork level and then identifies and utilizes some significant subnetworks. It has been shown that such a network-based approach not only improves predictive performance and reproducibility, but also sheds biological insights into molecular mechanisms underlying the clinical outcome. However, the above method is largely heuristic without a formal statistical framework; more importantly, it involves a random search over subnetworks, leading to possibly different results from different runs with no guarantee of the optimality of the final result. Because of the ever-increasing popularity of penalization methods for high-dimensional data, we propose a novel network-based penalty to be used with the hinge loss, leading to a network-based support vector machine. While maintaining some desirable properties of support vector machine (SVM) with the hinge loss function, the network-based penalty directly integrates a biological network to realize more effective variable selection, as compared with generic methods, such as the standard SVM (STD-SVM) or *L*_1_-penalized SVM (L1-SVM).

The support vector machine (SVM) is one of the most popular supervised learning techniques with wide-ranging applications [9,10]. In particular, previous studies have demonstrated its superior performance in gene expression data analysis, especially its ability to handle high dimensional data [11,12]. Nevertheless, with categorical predictors, both the STD-SVM and the L1-SVM may have some shortcomings. Zou and Yuan [13] applied the concept of grouped variable selection and developed an *F*_∞_-norm penalized SVM to realize simultaneous selection/elimination of all the features derived from the same categorical factor (or a group of variables). Their numerical examples showed that the *F*_∞_-norm SVM outperformed the L1-SVM in factor-wise variable selection. We extend the idea of variable grouping to gene networks: rather than grouping all the dummy variables created from the same categorical factor, we treat two neighboring genes in a network as one group. The network-based penalty is constructed as the sum of the *F*_∞_-norms being applied to the groups of neighboring-gene pairs. With the hinge loss penalized by such a network-based penalty as our objective function, we obtain our network-based SVM. The later sections are organized as follows. We begin with a brief review of the SVM, and then introduce our proposed network-based SVM. We evaluate its performance by simulation studies in both low dimensional and high dimensional data settings as well as two real data applications. The last section concludes the paper with a brief summary.

## Methods

### Existing methods

Suppose we have training data ${\left\{(x_{i},y_{i})\right\}}_{i=1}^{N}$ with *x*_*i *_∈ ℝ^*p *^and *y*_*i *_∈ {1, -1}. Define a hyperplane {*x *: *f*(*x*)= *x*^*T*^*β *+ *β*_0 _= 0}. The classification rule induced by *f *(*x*) is *sign *[$\hat{f}$(*x*)]. SVM searches for such a hyperplane $\hat{f}(x)=x^{T}\hat{\beta }+{\hat{\beta }}_{0}$ that maximizes the margin between the training data points for class 1 and class -1:

(1) $$\begin{array}{c} \underset{\beta ,\beta_{0}}{\max}\frac{1}{{\left\|\beta \right\|}_{2}}\\ \begin{array}{ccc} \text{subject to} & y_{i}(x_{i}^{T}\beta +\beta_{0})\geq 1-\xi_{i}, & \forall i \end{array}\\ \begin{array}{cc} \xi_{i}\geq 0, & \sum_{i=1}^{N}\xi_{i}\leq C \end{array} \end{array}$$

where *ξ*_*i *_are slack variables, and *C *is a tuning parameter to be determined. The STD-SVM has an equivalent *hinge loss + penalty *formulation as an optimization problem [13-15]:

(2) $$\underset{\beta_{0},\beta }{\min}\left\{\sum_{i=1}^{N}{\left[1-y_{i}(x_{i}^{T}\beta +\beta_{0})\right]}_{+}+\lambda {\left\|\beta \right\|}_{2}^{2}\right\}$$

where the subscript "+" denotes the positive part, i.e., *z*_+ _= *max*{*z*, 0}, ${\left\|\beta \right\|}_{2}^{2}=\sum_{k=1}^{p}{\left|\beta_{k}\right|}^{2}$, and *λ *is the tuning parameter. The solution to (1) is the same as that to (2).

The above STD-SVM forces all nonzero coefficient estimates, which leads to the problem of its inability to conduct variable selection. The L1-SVM was proposed to accomplish the goal of variable selection. It can be formulated as

(3) $$\underset{\beta_{0},\beta }{\min}\left\{\sum_{i=1}^{N}{\left[1-y_{i}(x_{i}^{T}\beta +\beta_{0})\right]}_{+}+\lambda {\left\|\beta \right\|}_{1}\right\}$$

where ${\left\|\beta \right\|}_{1}=\sum_{k=1}^{p}\left|\beta_{k}\right|$. The L1-SVM wins over the STD-SVM when the true model is sparse, while the STD-SVM is preferred if there are not many redundant noise features [16].

Zou and Yuan [13] pointed out the shortcoming of the *L*_1_-norm penalty: even though it encourages parsimonious models, it fails to guarantee successful models in cases of categorical predictors due to the fact that each dummy variable is selected independently. They applied the concept of grouped variable selection and proposed an *F*_∞_-norm SVM to realize simultaneous selection/elimination of features derived from the same factor so as to accomplish automatic factor-wise variable selection. Suppose we have *G *factors *F*_1_,...,*F*_*G*_. From each factor *F*_*g*_, we generate a feature vector $x_{\left(g\right)}={\left(x_{1}^{\left(g\right)},\cdots ,x_{j}^{\left(g\right)},\cdots ,x_{n_{g}}^{\left(g\right)}\right)}^{T}$.

Correspondingly we have the coefficient vector $\beta_{\left(g\right)}={\left(\beta_{1}^{\left(g\right)},\cdots ,\beta_{j}^{\left(g\right)},\cdots ,\beta_{n_{g}}^{\left(g\right)}\right)}^{T}$. Therefore,

(4) $$f(x)=x^{T}\beta +\beta_{0}=\sum_{g=1}^{G}x_{\left(g\right)}^{T}\beta_{\left(g\right)}+\beta_{0}$$

Define the *F*_∞_-norm of *F*_*g *_as

(5) $${\left\|F_{g}\right\|}_{\infty }={\left\|\beta_{\left(g\right)}\right\|}_{\infty }=\underset{j\in \{1,\cdots ,n_{g}\}}{\max}\{|\beta_{j}^{\left(g\right)}|\}$$

The *F*_∞_-norm SVM is formulated as

(6) $$\underset{\beta_{0},\beta }{\min}\left\{\sum_{i=1}^{N}{\left[1-y_{i}\left(\sum_{g=1}^{G}x_{i,(g)}^{T}\beta_{\left(g\right)}+\beta_{0}\right)\right]}_{+}+\lambda \sum_{g=1}^{G}{\left\|\beta_{\left(g\right)}\right\|}_{\infty }\right\}$$

The most noteworthy property of the *F*_∞_-norm SVM is its guarantee of sparsity at the factor level. Due to the singularity property of the infinity norm: || *β*_(*g*) _||_∞ _is not differentiable at *β*_(*g*) _= 0, *β*_(*g*) _will be exactly zero if the regularization parameter *λ *is properly chosen [13]. Therefore, the *F*_∞_-norm SVM automatically eliminates factors that are completely irrelevant to the response, and thus achieves the goal of factor-wise selection. The empirical evidence shows that the *F*_∞_-norm SVM often outperforms both the L1-SVM and the STD-SVM.

### New method

Biological observations reveal that neighboring genes in a network tend to function together in biological processes. To incorporate this prior information, a network-based SVM for binary classification is proposed to facilitate generating models that extract more biological insight from gene expression data. The penalty term that characterizes the network structure can be specified by implanting the *F*_∞_-norm into the context of known functional interrelationships among genes by considering each pair of the functionally related genes as one group.

Consider a gene network with *S *denoting the set of all edges, i.e., the pair of connected genes.

*S *= {(*j*_1_, *j*_2_) : gene *j*_1 _and gene *j*_2 _are connected}

Define *w*_*k *_as some weight for gene *k*. For example, *w*_*k *_= $\sqrt{d_{k}}$ where *d*_*k *_is the number of direct neighbors of gene *k*, or *w*_*k *_= *d*_*k*_, or simply *w*_*k *_= 1 for all genes. We propose a novel penalty in the form of

(7) $$\sum_{(j_{1},j_{2})\in S}\max\left\{\frac{\left|\beta_{j_{1}}\right|}{w_{j_{1}}},\frac{\left|\beta_{j_{2}}\right|}{w_{j_{2}}}\right\}$$

Thus the network-based SVM solves the optimization problem as follows.

(8) $$\underset{\beta_{0},\beta }{\min}\left\{\sum_{i=1}^{N}{\left[1-y_{i}(x_{i}^{T}\beta +\beta_{0})\right]}_{+}+\lambda \sum_{(j_{1},j_{2})\in S}\max\left\{\frac{\left|\beta_{j_{1}}\right|}{w_{j_{1}}},\frac{\left|\beta_{j_{2}}\right|}{w_{j_{2}}}\right\}\right\}$$

Four properties of the penalty term are noteworthy. First, the regularization is performed at the level of grouped genes with each group containing two neighboring genes in the network. In the case of penalized linear regression, it has been proven that this penalty achieves the goal of eliminating both $\beta_{j_{1}}$ and $\beta_{j_{2}}$ simultaneously if (*j*_1_, *j*_2_) ∈ *S *[17]. The automatic selection of grouped features is due to the singularity of function max{|*a*|, |*b*|} [13]. This formulation satisfies our assumption that neighboring genes tend to (or not to) contribute to the same biological process at the same time. Second, the choice of the weight depends on the goal of shrinkage and influences the predictive performance. Consider a network comprised of several subnetworks, each with one regulator and ten target genes. Because of the singularity of function max(|*a*|, |*b*|) at *a *= *b*, the weighted penalty in the context of penalized regression, encourages $\left|\beta_{j_{1}}\right|/w_{j_{1}}=\left|\beta_{j_{2}}\right|/w_{j_{2}}$[17]. Here we examine three weight functions in particular: *w*_*k *_= 1, *w*_*k *_= $\sqrt{d_{k}}$, and *w*_*k *_= *d*_*k*_, where gene *k *has *d*_*k *_direct neighbors. The new method encourages $\left|\beta_{j_{1}}\right|=\left|\beta_{j_{2}}\right|$ if *w*_*k *_= 1, $\frac{\left|\beta_{j_{1}}\right|}{\sqrt{d_{j_{1}}}}=\frac{\left|\beta_{j_{2}}\right|}{\sqrt{d_{j_{2}}}}$ if *w*_*k *_= $\sqrt{d_{k}}$, and $\frac{\left|\beta_{j_{1}}\right|}{d_{j_{1}}}=\frac{\left|\beta_{j_{2}}\right|}{d_{j_{2}}}$ if *w*_*k *_= *d*_*k*_. Therefore, heavier weights (from *w*_*k *_= 1, *w*_*k *_= $\sqrt{d_{k}}$, to *w*_*k *_= *d*_*k*_) favor genes with more direct neighbors to have larger coefficient estimates; in other words, heavier weights relax the shrinkage effect for those regulators, which are known to be biologically more important. Due to this property, the choice of a heavy weight, as a simple strategy, enables us to alleviate the bias in the coefficient estimates from the penalization method and possibly improve the *p *predictive performance. Our default weight is *w*_*k *_= $\sqrt{d_{k}}$. The weight, considered as another tuning parameter, can be determined from cross-validation or an independent validation data set, though we do not consider it here. Third, the penalty term, under certain conditions, tends to encourage a grouping effect, where highly correlated predictors tend to have similar coefficient estimates [17-20]. Fourth, the penalty is linear, which allows the solution to be found by the linear programming (LP) technique that is computationally convenient.

As usual, the fitted classifier is $\hat{f}(x)={\hat{\beta }}_{0}+x^{T}\hat{\beta }$, and the classification rule is *sign*($\hat{f}$(*x*)). We employ LP to obtain the solutions to (8) by

(9) $$\underset{\beta_{0},\beta }{\min}\left(\sum_{i=1}^{N}\xi_{i}+\lambda \sum_{(j_{1},j_{2})\in S}M_{\left(j_{1},j_{2}\right)}\right)$$

subject to

(10) $$\begin{array}{cc} y_{i}(\beta_{0}^{+}-\beta_{0}^{-}+x_{i}^{T}(\beta^{+}-\beta^{-}))\geq 1-\xi_{i}, & \begin{array}{cc} \xi_{i}\geq 0 & \forall i \end{array}\\ \begin{array}{cc} \frac{\beta_{j}^{+}}{w_{j}}+\frac{\beta_{j}^{-}}{w_{j}}\leq M_{\left(j_{1},j_{2}\right)}, & j=j_{1},j_{2} \end{array} & \forall (j_{1},j_{2})\in S\\ \begin{array}{ccc} \beta_{j}^{+}\geq 0, & \beta_{j}^{-}\geq 0, & j=j_{1},j_{2} \end{array} & \forall (j_{1},j_{2})\in S \end{array}$$

where

(11) $$\begin{array}{cc} \xi_{i}={\left[1-y_{i}\left(\sum_{i=1}^{N}x_{i}^{T}\beta +\beta_{0}\right)\right]}_{+}, & i=1,2,...,N \end{array}$$

(12) $$M_{\left(j_{1},j_{2}\right)}=\max\left\{\frac{\left|\beta_{j_{1}}\right|}{w_{j_{1}}},\frac{\left|\beta_{j_{2}}\right|}{w_{j_{2}}}\right\}$$

and $\beta_{j}=\beta_{j}^{+}-\beta_{j}^{-}$, in which $\beta_{j}^{+}$ and $\beta_{j}^{-}$ denote the positive and negative parts of *β*_*j*_. The calculation of the new method can be easily implemented by the R package lpsolve, so is the computation of the L1-SVM. The R package e1071 (with linear kernel) is used to obtain the solution to the STD-SVM.

## Results and discussion

### Simulation

We conducted several simulation studies to numerically evaluate the performance of the network-based SVM along with the STD-SVM and L1-SVM. The simulation setups were similar to those in [18]. We started from a simple network consisting of 5 subnetworks, each having a regulator gene *t *(*t *= 1,...,5) that regulated 10 target genes, leading to a total of 55 genes (*p *= 55). We assumed that two out of the five subnetworks were informative; that is, the coefficients of 22 genes were nonzero and thus informative to the outcome, while the remaining 33 noise genes had no effect on the outcome. We generated a simulated data set by the following steps:

• Generate the expression level of regulator gene *t*, *X*_*t *_~ *N *(0, 1), *t *= 1,..., 5, independently.

• Assume that the expression level of regulator gene *t *and each of its regulated genes follow a bivariate normal distribution with correlation 0.7. Thus, the expression level of each target gene regulated by gene *t*, $X_{l}^{\left(t\right)}$ ~ *N*(0.7*X*_*t*_, 0.51), *l *= 1,..., 10 and *t *= 1,..., 5.

• Generate the outcome *Y *from a logistic regression model: Logit (*Pr*(*Y *= 1|*X*)) = *X*^*T*^*β *+ *β*_0_, *β*_0_= 2, where *X *is the vector of the expression levels of all the genes, and coefficient vector $\beta =(\beta_{1}^{\left(1\right)},...,\beta_{10}^{\left(1\right)},...,\beta_{1}^{\left(5\right)},...,\beta_{10}^{\left(5\right)})$.

Four sets of true coefficients, *β *'s, were specified to reflect four scenarios:

1. $\beta =(5,\underset{10}{\underset{︸}{\frac{5}{\sqrt{10}},\cdots ,\frac{5}{\sqrt{10}}}},-5,\underset{10}{\underset{︸}{\frac{-5}{\sqrt{10}},\cdots ,\frac{-5}{\sqrt{10}}}},0,\cdots ,0).$.

The effect of one informative subnetwork was the same as the other in magnitude but with an opposite direction.

2. $\beta =(5,\underset{10}{\underset{︸}{\frac{5}{\sqrt{10}},\cdots ,\frac{5}{\sqrt{10}}}},3,\underset{10}{\underset{︸}{\frac{3}{\sqrt{10}},\cdots ,\frac{3}{\sqrt{10}}}},0,\cdots ,0).$.

Both informative subnetworks had positive effects but in different magnitudes.

3. $\beta =(5,\underset{7}{\underset{︸}{\frac{5}{\sqrt{10}},\cdots ,\frac{5}{\sqrt{10}}}},\frac{-5}{\sqrt{10}},\frac{-5}{\sqrt{10}},\frac{-5}{\sqrt{10}},3,\underset{7}{\underset{︸}{\frac{3}{\sqrt{10}},\cdots ,\frac{3}{\sqrt{10}}}},\frac{-3}{\sqrt{10}},\frac{-3}{\sqrt{10}},\frac{-3}{\sqrt{10}}0,\cdots ,0).$.

Target genes in the same informative subnetworks had both positive and negative effects.

4. $\beta =(5,\underset{6}{\underset{︸}{\frac{5}{\sqrt{10}},\cdots ,\frac{5}{\sqrt{10}}}},\underset{4}{\underset{︸}{\frac{-5}{\sqrt{10}},\cdots ,\frac{-5}{\sqrt{10}}}},-3,\underset{6}{\underset{︸}{\frac{-3}{\sqrt{10}},\cdots ,\frac{-3}{\sqrt{10}}}},\underset{4}{\underset{︸}{\frac{3}{\sqrt{10}},\cdots ,\frac{3}{\sqrt{10}}}},0,\cdots ,0).$.

It was similar to but more extreme than scenario 3.

Five methods, STD-SVM, L1-SVM, and network-based SVM with *w*_*k *_= 1, *w*_*k *_= $\sqrt{d_{k}}$, and *w*_*k *_= *d*_*k*_, were compared based on the results averaged over 100 runs under each of the above four scenarios. For each run, 100 observations were simulated as training data to build a classifier (with any given *λ*), another 100 for tuning the regularization parameter *λ*, and the last 10,000 as test data. Each predictor was normalized to have mean 0 and standard deviation 1. Given any value of *λ*, we obtained the coefficient estimates from the training set, then applied the classifier to the tuning set to find the classification error. We searched for $\hat{\lambda }$, from a wide range of prespecified values, which produced the smallest classification error. The classifier corresponding to $\hat{\lambda }$ was identified as the fitted classifier $\hat{f}$. Then we applied $\hat{f}$ to the test set and calculated the test error, the number of misclassifications divided by the test sample size. Table 1 reports the mean classification error of the test set and its standard error (SE in parentheses), the standard deviation of the classification errors divided by the square root of the number of runs, for each method over 100 runs under each scenario. To evaluate each method's ability to select informative genes, we examined the false negatives, defined as the number of informative genes whose coefficients were estimated to be zero. In addition, we also considered a smaller sample size: we repeated the entire process with 50 training data points, 50 tuning data points, and again 10,000 test data points. The network-based SVM is named as "New" in the table.

**Table 1:** Simulation results for *p *= 55. The simulation results were averaged over 100 runs for *p *= 55 (22 informative and 33 noise genes).

		Test Error (SE)		# False Negative (SE)		Model Size (SE)	
Scenario	Method	*n *= 50	*n *= 100	*n *= 50	*n *= 100	*n *= 50	*n *= 100
1	STD	0.122 (0.002)	0.096 (0.001)	0.0 (0.0)	0.0 (0.0)	55.0 (0.0)	55.0 (0.0)
	L1	0.134 (0.003)	0.094 (0.002)	13.1 (0.3)	10.9 (0.4)	12.3 (0.6)	15.3 (0.7)
	New (*w *= 1)	0.156 (0.003)	0.105 (0.002)	9.3 (0.4)	2.4 (0.3)	17.0 (0.6)	24.3 (0.6)
	New (*w *= $\sqrt{d}$)	0.111 (0.003)	0.068 (0.002)	1.0 (0.3)	0.1 (0.1)	24.7 (0.5)	25.1 (0.4)
	New (*w *= *d*)	0.081 (0.002)	0.059 (0.002)	0.0 (0.0)	0.0 (0.0)	28.6 (0.8)	28.2 (0.8)

2	STD	0.121 (0.002)	0.099 (0.001)	0.0 (0.0)	0.0 (0.0)	55.0 (0.0)	55.0 (0.0)
	L1	0.133 (0.003)	0.096 (0.001)	13.6 (0.3)	11.1 (0.4)	11.4 (0.5)	15.1 (0.7)
	New (*w *= 1)	0.156 (0.003)	0.105 (0.002)	9.6 (0.4)	3.9 (0.3)	16.3 (0.7)	24.7 (0.6)
	New (*w *= $\sqrt{d}$)	0.121 (0.003)	0.075 (0.002)	3.0 (0.4)	0.3 (0.1)	22.3 (0.6)	25.2 (0.5)
	New (*w *= *d*)	0.083 (0.002)	0.064 (0.002)	0.0 (0.0)	0.0 (0.0)	28.6 (0.8)	29.0 (0.8)

3	STD	0.162 (0.002)	0.138 (0.001)	0.0 (0.0)	0.0 (0.0)	55.0 (0.0)	55.0 (0.0)
	L1	0.166 (0.003)	0.131 (0.001)	13.9 (0.2)	11.0 (0.3)	11.2 (0.5)	16.6 (0.7)
	New (*w *= 1)	0.177 (0.003)	0.140 (0.002)	12.4 (0.4)	7.7 (0.4)	13.5 (0.6)	19.9 (0.8)
	New (*w *= $\sqrt{d}$)	0.164 (0.003)	0.127 (0.002)	4.4 (0.5)	1.2 (0.3)	21.5 (0.6)	26.3 (0.7)
	New (*w *= *d*)	0.137 (0.003)	0.114 (0.001)	0.4 (0.2)	0.1 (0.1)	29.8 (0.9)	33.2 (0.9)

4	STD	0.189 (0.002)	0.157 (0.002)	0.0 (0.0)	0.0 (0.0)	55.0 (0.0)	55.0 (0.0)
	L1	0.186 (0.002)	0.155 (0.002)	14.2 (0.3)	10.5 (0.3)	11.5 (0.6)	18.1 (0.8)
	New (*w *= 1)	0.198 (0.003)	0.160 (0.002)	13.8 (0.3)	8.6 (0.4)	11.8 (0.5)	20.9 (0.9)
	New (*w *= $\sqrt{d}$)	0.190 (0.003)	0.147 (0.002)	7.2 (0.6)	1.8 (0.4)	18.8 (0.7)	30.1 (0.9)
	New (*w *= *d*)	0.163 (0.002)	0.139 (0.002)	0.2 (0.2)	0.03 (0.03)	32.2 (1.0)	34.8 (1.0)

According to our simulation setups, the correct weight function should be *w *= $\sqrt{d}$. However, we find that the new method with *w *= *d *overwhelmingly beat all other methods in all the setups. It consistently made the most accurate classifications and missed no informative genes. The new method with *w *= $\sqrt{d}$ performed the second best: in most cases, it improved the classification accuracy over STD-SVM and L1-SVM; and under all the settings, it produced models that identified more informative genes than the L1-SVM. In contrast, *w *= 1 did not bring much gains over the STD-SVM or the L1-SVM. The L1-SVM led to models that were too sparse, missing about 14 and 11 informative genes for *n *= 50 and *n *= 100 respectively. The superior performance and the larger model size of the heavy weight (*w *= *d*) compared with its counterparts (*w *= 1 and *w *= $\sqrt{d}$) is presumably due to its relaxation of the shrinkage effect. The penalization methods shrink the $\hat{\beta }$ toward zero by imposing the constraints (the penalty term) and therefore introduces bias to $\hat{\beta }$. By grouping neighboring genes, the new method encourages the pairwise weighted absolute coefficients to be equal. Therefore, a heavy weight leads to larger |$\hat{\beta }$| for regulator genes. By choosing a heavier weight, we may overcome over-shrinkage, alleviate biases, and achieve better classification accuracy to some extent at the expense of model sparsity. As shown by Table 2, *w *= *d *produced the largest |$\hat{\beta }$| for regulators than its two counterparts. The L1-SVM estimates were treated as a yardstick for comparison as to provide an idea of the extent of shrinkage by each weight function. For example, *w *= 1 and *w *= $\sqrt{d}$ overly shrank all the regulators under all scenarios as compared with the L1-SVM estimates. Note that the binary outcome *Y *was generated from a logistic regression model while $\hat{\beta }$ was estimated from a linear model, hence *E*($\hat{\beta }$) may be different from *β *even for an unbiased estimator $\hat{\beta }$ of the linear model.

**Table 2:** Coefficient estimates of selected informative genes for *p *= 55 and *n *= 100. The mean and the standard deviation (SD) of the coefficient estimates for selected informative genes were calculated from 100 runs.

		L1		New (*w *= 1)		New (*w *= $\sqrt{d}$)		New (*w *= *d*)	
Scenario	*β*	Mean	SD	Mean	SD	Mean	SD	Mean	SD
1	*β*_1 _= 5	0.53	0.29	0.04	0.04	0.27	0.26	0.67	0.35
	$\beta_{1}^{\left(1\right)}=\frac{5}{\sqrt{10}}$	0.11	0.17	0.14	0.15	0.10	0.10	0.07	0.08
	*β*_2 _= -5	-0.55	0.30	-0.04	0.05	-0.28	0.32	-0.68	0.35
	$\beta_{1}^{\left(2\right)}=\frac{-5}{\sqrt{10}}$	-0.08	0.15	-0.18	0.15	-0.11	0.09	-0.08	0.08

2	*β*_1 _= 5	0.76	0.33	0.09	0.06	0.34	0.16	0.91	0.40
	$\beta_{1}^{\left(1\right)}=\frac{5}{\sqrt{10}}$	0.09	0.14	0.20	0.14	0.14	0.11	0.09	0.08
	*β*_2 _= 3	0.29	0.23	0.01	0.03	0.15	0.10	0.48	0.23
	$\beta_{1}^{\left(2\right)}=\frac{3}{\sqrt{10}}$	0.08	0.12	0.11	0.13	0.07	0.08	0.04	0.04

3	*β*_1 _= 5	0.51	0.39	0.03	0.07	0.41	0.70	0.95	0.34
	$\beta_{1}^{\left(1\right)}=\frac{5}{\sqrt{10}}$	0.22	0.21	0.24	0.19	0.20	0.17	0.13	0.11
	$\beta_{8}^{\left(1\right)}=\frac{-5}{\sqrt{10}}$	-0.01	0.07	-0.01	0.11	-0.03	0.21	-0.04	0.12
	*β*_2 _= 3	0.26	0.27	0.01	0.04	0.15	0.30	0.52	0.27
	$\beta_{1}^{\left(2\right)}=\frac{3}{\sqrt{10}}$	0.09	0.13	0.13	0.16	0.12	0.16	0.07	0.11
	$\beta_{8}^{\left(2\right)}=\frac{-3}{\sqrt{10}}$	0.001	0.07	0.004	0.06	0.01	0.05	-0.01	0.07

4	*β*_1 _= 5	0.40	0.38	0.03	0.06	0.48	0.80	0.97	0.43
	$\beta_{1}^{\left(1\right)}=\frac{5}{\sqrt{10}}$	0.27	0.26	0.32	0.25	0.30	0.23	0.20	0.20
	$\beta_{7}^{\left(1\right)}=\frac{-5}{\sqrt{10}}$	-0.04	0.12	-0.02	0.14	-0.11	0.24	-0.09	0.16
	*β*_2 _= -3	-0.23	0.29	-0.004	0.01	-0.21	0.45	-0.56	0.30
	$\beta_{1}^{\left(2\right)}=\frac{-3}{\sqrt{10}}$	-0.15	0.20	-0.16	0.19	-0.17	0.19	-0.09	0.13
	$\beta_{7}^{\left(2\right)}=\frac{3}{\sqrt{10}}$	0.03	0.08	-0.002	0.10	0.05	0.18	0.06	0.15

Next, we evaluated the performance of the new method for high-dimensional data with large *p*. We used the setup of 50 observations for training, 50 for tuning, and 10,000 for test data. We assumed that (1) the network was composed of either 50 or 100 subnetworks, each having one gene regulating 10 target genes; (2) the first 2 subnetworks were informative resulting in 22 informative genes; (3) the rest of the genes had no effect on the outcome, leading to 528 noise genes when *p *= 550 and 1,078 noise genes when *p *= 1, 100; and (4) the true *β *was specified as in scenario 3. Table 3 shows the simulation results averaged over 100 runs. Again, we see the gains from using a heavy weight (*w *= *d*). It prevailed over all the other methods in making accurate classifications and selecting informative genes. The *w *= $\sqrt{d}$ ranked the second. However, *w *= *d *generated models much larger than those from other methods except STD-SVM. In this case, the performance of *w *= 1 is no better than L1-SVM possibly due to over shrinkage of the effects of the regulator genes.

**Table 3:** Simulation results for *p *= 550 or 1, 100. The simulation results were averaged over 100 runs for *p *= 550 or 1, 100 (22 informative and either 528 or 1,078 noise genes).

	Test Error (SE)		# False Negative (SE)		Model Size (SE)	
Method	*p *= 550	*p *= 1, 100	*p *= 550	*p *= 1, 100	*p *= 550	*p *= 1, 100
STD	0.305 (0.003)	0.354 (0.002)	0.0 (0.0)	0.0 (0.0)	550 (0.0)	1,100 (0.0)
L1	0.218 (0.004)	0.235 (0.004)	16.6 (0.2)	17.1 (0.2)	16.1 (1.0)	19.2 (1.2)
New (*w *= 1)	0.232 (0.003)	0.255 (0.004)	14.9 (0.3)	15.6 (0.3)	20.7 (1.1)	22.6 (1.4)
New (*w *= $\sqrt{d}$)	0.202 (0.004)	0.221 (0.004)	5.7 (0.5)	6.7 (0.6)	32.6 (1.5)	34.6 (1.9)
New (*w *= *d*)	0.170 (0.003)	0.180 (0.004)	0.7 (0.3)	1.3 (0.4)	82.6 (5.4)	98.9 (7.2)

### Applications to microarray data

To evaluate its performance in the real world, we applied the new method to two microarray gene expression data sets related to the Parkinson's disease (PD) [21] and breast cancer metastasis (BC) [1,4] respectively.

#### Parkinson's disease

The data set includes the Parkinson's disease status and the expression levels of 22,283 genes from 105 patients (50 cases and 55 controls) [22]. We used the same network structure as [18]. The network combines 33 Kyoto Encyclopedia of Genes and Genomes (KEGG) regulatory pathways and contains a total of 1,523 genes and 6,865 edges. The data were randomly split into training (40 observations), tuning (20 observations), and test (45 observations) sets. The expression level of each gene was normalized to have mean 0 and standard deviation 1 across samples. The tuning parameter was identified from the tuning set and the performance of the method was evaluated on the test set by the mean classification error and its standard error averaged over 10 runs. Five methods were compared: STD-SVM, L1-SVM, network-based SVM with *w *= 1, *w *= $\sqrt{d}$, and *w *= *d*. To obtain a final model based on the new method with *w *= $\sqrt{d}$, we combined, for each run, the previous tuning and test data as the new tuning set leading to a sample size as large as 65 observations, on which the classification errors were calculated for wide-ranging values of the tuning parameter. Then after 10 runs, we had an averaged classification error corresponding to each tuning parameter value. The value that generated the minimal averaged error was the one we selected to fit the final model to all the data. Note that the classification error rate from the final model was likely to be biased due to the double use of the data for training/tuning and test; the main purpose of fitting the final model was to see the selected genes at the end.

First, we focused on the 1,070 genes that appeared in the network with the largest variations of expression levels (i.e., SD of expression levels across the 105 samples ≥ 15). According to the KEGG pathway of Parkinson's disease [23], 20 genes play a role in the disease progression, five of which (*UBE1, PARK2, UBB, SEPT5*, and *SNCAIP*) belong to the 1,070 genes. In addition to the classification error, we added two additional criteria for method comparison: the number of disease genes identified, and the number of genes identified. Table 4 shows that STD-SVM made the most accurate classification, even though the difference with other methods was perhaps non-significant. The *w *= *d *ranked the second in predictive performance while produced a model including 70.6 genes on average. In this case, the *w *= $\sqrt{d}$ gained advantage: it selected more disease genes by a relatively sparse model with a classification error non-significantly larger than STD-SVM. From the 1,070 genes, with the final model the new method identified 75 genes including one disease gene.

**Table 4:** Parkinson's disease data: 1,070 genes. A total of 1,070 genes with SD of expression levels across the 105 samples ≥ 15 had network information. The classification error, number of selected disease genes, number of selected genes, and their standard errors (SE in parentheses) were obtained by averaging over 10 runs. Five disease genes were *UBE1, PARK2, UBB, SEPT5*, and *SNCAIP*.

Method	Error	# Disease Genes	# Genes
STD	0.424 (0.016)	5.0 (0.0)	1,070.0 (0.0)
L1	0.464 (0.021)	0.1 (0.1)	19.2 (3.8)
New (*w *= 1)	0.476 (0.015)	0.1 (0.1)	24.9 (4.3)
New (*w *= $\sqrt{d}$)	0.480 (0.026)	0.2 (0.1)	30.6 (5.2)
New (*w *= *d*)	0.451 (0.028)	0.0 (0.0)	70.6 (14.1)

Final Model	-	1.0	75.0

**Figure 1:**
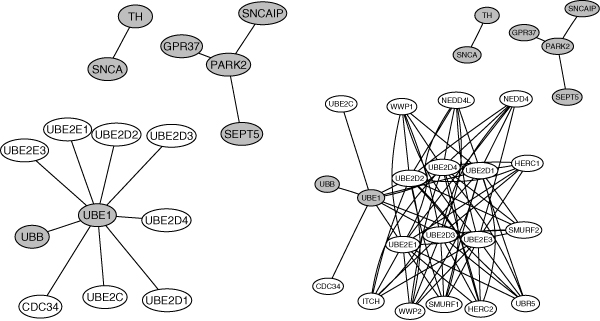
**Parkinson's disease gene subnetworks**. Left: PD-1nb-net, including 8 Parkinson disease genes (gray) and their 8 direct neighbors (white). Right: PD-2nb-net, including 8 Parkinson disease genes (gray), their 8 direct and 10 second-order neighbors (white).

Next, to better integrate the biological observation of the KEGG pathway and the known network structure of [18], we restricted our analysis to the first- and second-order-neighbors of the 8 disease genes on the Parkinson's disease KEGG pathway whose expression levels and network structure are available. The first-order-neighbor subnetwork (PD-1nb-net) was composed of the 8 disease genes and their 8 direct neighbors. The second-order-neighbor subnetwork (PD-2nb-net) comprised the PD-1nb-net as well as the direct neighbors of the 8 direct neighbors of the disease genes, leading to a total of 26 genes. Figure 1 displays the two subnetworks. We conducted the analysis in the same way as described above. The only difference resided in that this time only genes appearing in the PD-1nb-net and PD-2nb-net were included in the analysis. Table 5 shows the results.

**Table 5:** First- and second-order-neighbor subnetworks of Parkinson's disease data. The classification error, number of selected disease genes, number of selected genes, and their standard errors (SE in parentheses) were obtained by averaging over 10 runs. Eight disease genes were *UBE1, PARK2, UBB, SEPT5, SNCAIP, GPR37, TH*, and *SNCA*.

Network	Method	Error	# Disease Genes	# Genes
PD-1nb-net	STD	0.476 (0.023)	8.0 (0.0)	16.0 (0.0)
	L1	0.471 (0.017)	2.8 (0.7)	6.1 (1.5)
	New (*w *= 1)	0.462 (0.016)	3.4 (0.8)	7.3 (1.7)
	New (*w *= $\sqrt{d}$)	0.462 (0.014)	3.6 (0.7)	8.4 (1.5)
	New (*w *= *d*)	0.482 (0.015)	3.0 (1.2)	7.5 (2.1)
	Final Model	-	8.0	16.0

PD-2nb-net	STD	0.444 (0.016)	8.0 (0.0)	26.0 (0.0)
	L1	0.449 (0.017)	3.1 (0.5)	10.9 (2.1)
	New (*w *= 1)	0.464 (0.022)	5.3 (0.9)	13.2 (3.2)
	New (*w *= $\sqrt{d}$)	0.447 (0.023)	6.1 (0.8)	13.7 (2.7)
	New (*w *= *d*)	0.433 (0.016)	6.2 (0.9)	20.0 (2.5)
	Final Model	-	8.0	26.0

We see the gains from employing the new method when narrowing down our focus on the PD-1nb-net and PD-2nb-net. For the PD-1nb-net, *w *= 1 and *w *= $\sqrt{d}$ performed equally well. They had the smallest classification error and identified one more disease gene through a model slightly larger than the one obtained from L1-SVM. The new method with *w *= *d *won over in the case of PD-2nb-net with the best accuracy and most selected disease genes. The *w *= $\sqrt{d}$ ranked the second in terms of the prediction accuracy while detecting 3 more disease genes by a model with 3 more genes than that of the L1-SVM. This means that the new method was able to identify more clinically relevant genes while keeping the same number of noise genes in the model as L1-SVM. In both subnetworks, the final models included all the genes.

#### Breast cancer metastasis

The breast cancer metastasis data set [1,4] contains expression levels of 8,141 genes for 286 patients, 106 of whom were detected to develop metastasis within a 5-year follow-up after surgery. *TP53*, *BRCA1*, and *BRCA2 *are three human genes that belong to the class of tumor suppressor genes, which are known to prevent uncontrolled cell proliferation, and to play a critical role in repairing the chromosomal damage. Certain mutations of these genes lead to increasing risk of breast cancer. We explored the protein-protein interaction (PPI) network previously used by [1]. The PPI network comprises 57,235 interactions among 11,203 proteins, obtained by assembling various sources of experimental data and curation of the literature [1]. We confined our analysis to the direct or first-order neighbors (BC-1nb-net) of the three cancer genes, and the subnetwork composed of two parts (BC-2nb-net): the direct neighbors of *TP53*, and the second-order neighbors of *BRCA1 *and *BRCA2*. We fit the final model and compared the four methods in terms of classification error, cancer genes selection, and model sparsity. The cancer genes are the 227 known or putative cancer genes with estimated mutation frequencies in cancer samples ([1]). A total of 294 genes that fell into the BC-1nb-net had observed expression levels, among which were 40 cancer genes and 7 cancer genes (*ABL1, JAK2, p53, PTEN, p14ARF, PTCH*, and *RB*) with mutation frequencies larger than 0.10. The BC-2nb-net was composed of 2,070 genes, 1,718 of them with observed expression levels, including 107 cancer genes. Besides the 7 included in BC-1nb-net, 7 additional cancer genes (*ACH, APC, EGFR, KIT, NICD, RAS*, and *CTNNB1*) that had mutation frequencies larger than 0.10 belonged to BC-2nb-net.

For BC-1nb-net, *w *= *d *had the advantage in selecting cancer genes and those with large mutant frequencies (Table 6). The *w *= $\sqrt{d}$ detected more clinically relevant genes by a sparser model while reaching a comparable classification error rate to that of L1-SVM. Even though the final model was parsimonious, it included 4 cancer genes, one of which had a large mutation frequency. For BC-2nb-net, the new method with *w *= $\sqrt{d}$ detected more cancer genes with equally accurate predictions while maintaining a sparse model compared with L1-SVM. The final model included only 23 genes out of 1,718, two of which were cancer genes with one having a large mutation frequency.

**Table 6:** Subnetworks of breast cancer data. The BC-1nb-net/BC-2nb-net had 294/1,718 genes in total including 40/107 cancer genes, and 7/14 cancer genes with mutation frequencies larger than 0.10. The classification error, number of selected cancer genes with mutation frequencies larger than 0.10 (CA-LMF), number of selected cancer genes (CA), number of selected genes, and their standard errors (SE in parentheses) were obtained by averaging over 10 runs.

Network	Method	Error	# CA-LMF	# CA	# Genes
BC-1nb-net	STD	0.371 (0.014)	7.0 (0.0)	40.0 (0.0)	294.0 (0.0)
	L1	0.357 (0.014)	0.3 (0.2)	4.6 (0.8)	32.3 (4.8)
	New (*w *= 1)	0.360 (0.014)	0.4 (0.2)	3.6 (1.1)	25.0 (7.0)
	New (*w *= $\sqrt{d}$)	0.366 (0.012)	0.6 (0.3)	4.7 (1.2)	27.2 (5.2)
	New (*w *= *d*)	0.399 (0.012)	1.2 (0.2)	7.8 (1.7)	40.2 (6.5)
	Final Model	-	1.0	4.0	14.0

BC-2nb-net	STD	0.351 (0.014)	14.0 (0.0)	107.0 (0.0)	1,718.0 (0.0)
	L1	0.360 (0.006)	0.0 (0.0)	2.4 (0.9)	42.9 (11.8)
	New (*w *= 1)	0.374 (0.011)	0.1 (0.1)	1.9 (0.5)	51.4 (12.6)
	New (*w *= $\sqrt{d}$)	0.360 (0.007)	0.2 (0.1)	2.5 (0.7)	41.7 (9.2)
	New (*w *= *d*)	0.385 (0.021)	0.3 (0.2)	0.7 (0.3)	34.2 (10.3)
	Final Model	-	1.0	2.0	23.0

## Conclusion

The advancement in the microarray technology has enriched the tool kit of researchers to decipher the complexity of disease mechanisms at the genomic level. Studies have been widely conducted to identify genetic markers to better the diagnostic classification and prognostic assessment, largely by ignoring biological knowledge on gene functions and treating individual genes equally and independently a priori. The downside of such an endeavor has been realized; for example, gene markers identified across similar patient cohorts for the same disease in such a way often lack consistency. As a viable alternative, the network-based approach has been gaining popularity. In addition to improving predictive performance and gene selection, the network-based approach extracts more biological insights from high-throughput gene expression data. Here we have proposed a network-based SVM, with a penalty term incorporating gene network information, as a practically useful classification tool for microarray data. Our simulation studies and two real data applications indicate that the proposed method is able to better identify clinically relevant genes and make accurate predictions.

## List of abbreviations used

SVM: support vector machine; STD-SVM: standard support vector machine; L1-SVM: L1-penalized support vector machine; LP: linear programming; PD: Parkinson's disease; BC: Breast cancer; KEGG: Kyoto Encyclopedia of Genes and Genomes; PPI: protein protein interaction

## Competing interests

The authors declare that they have no competing interests.

## Authors' contributions

YZ implemented the methods, did all the experiments and drafted the paper. XS and WP initiated the project. All participated in the writing of the article.
